# Readiness of the Belgian network of sentinel general practitioners to deliver electronic health record data for surveillance purposes: results of survey study

**DOI:** 10.1186/1471-2296-11-50

**Published:** 2010-06-25

**Authors:** Nicole Boffin, Nathalie Bossuyt, Katrien Vanthomme, Viviane Van Casteren

**Affiliations:** 1 Public Health and Surveillance, Scientific Institute of Public Health, Brussels, Belgium

## Abstract

**Background:**

In order to proceed from a paper based registration to a surveillance system that is based on extraction of electronic health records (EHR), knowledge is needed on the number and representativeness of sentinel GPs using a government-certified EHR system and the quality of EHR data for research, expressed in the compliance rate with three criteria: recording of home visits, use of prescription module and diagnostic subject headings.

**Methods:**

Data were collected by annual postal surveys between 2005 and 2009 among all sentinel GPs. We tested relations between four key GP characteristics (age, gender, language community, practice organisation) and use of a certified EHR system by multivariable logistic regression. The relation between EHR software package, GP characteristics and compliance with three quality criteria was equally measured by multivariable logistic regression.

**Results:**

A response rate of 99% was obtained. Of 221 sentinel GPs, 55% participated in the surveillance without interruption from 2005 onwards, i.e. all five years, and 78% were participants in 2009. Sixteen certified EHR systems were used among 91% of the Dutch and 63% of the French speaking sentinel GPs. The EHR software package was strongly related to the community and only one EHR system was used by a comparable number of sentinel GPs in both communities. Overall, the prescription module was always used and home visits were usually recorded. Uniform subject headings were only sometimes used and the compliance with this quality criterion was almost exclusively related to the EHR software package in use.

**Conclusions:**

The challenge is to progress towards a sentinel network of GPs delivering care-based data that are (partly) extracted from well performing EHR systems and still representative for Belgian general practice.

## Background

The Belgian network of sentinel general practitioners (GPs) was developed in 1979, drawing on experiences of the Weekly Returns Service of the Royal College of General Practitioners and the Dutch Sentinel Stations in the Netherlands [1]. In the first European study among similar networks, this type of sentinel surveillance was described as "a system that keeps a watchful eye on a sample of the population by supplying regular and standardized reports on the incidence and main epidemiological characteristics of specific diseases and procedures in primary health care" [2].

Nowadays, the importance of epidemiological research on health problems in primary care is well accepted and the characteristics of primary care based research networks have been described more than once [3-6]. In 2002-2003, site visits to seven "well-established surveillance networks" in primary care showed that four of them, among which the Belgian network, were still using paper forms [3]. Automatic data collection obtained from electronic medical records (EHR) was one of the recommendations of the inventory. Considerations and requests to shift to a paperless registration have been uttered since several years by both managers and members of the Belgian sentinel network. Proposals ranged from web-based data entry to automatic extraction of EHR data, either routinely collected or recorded purposively for the surveillance system. While EHR systems are primarily designed for clinical information management they might also be used for research. Opportunities and challenges of EHR data for research purposes have already been described [7-9].

In order to develop a surveillance system that is (partly) based on extraction of EHR, a substantial number of sentinel GPs should use a limited, manageable number of the 20 government-certified EHR systems on the Belgian market. Also, the well-guarded representativeness of the network should be preserved. Our annual surveys showed that age and sex distributions in the network of sentinel GP were highly similar to those of all Belgian GPs and those living in the Northern and Southern region [10].

At present there is no report providing an overview of the distribution and features of EHR systems in Belgian general practice and the functions used by GPs. The rate of use of certified EHR systems in the Belgian sentinel GPs is known by our annual surveys. But so far we did not analyze the pooled data set of all sentinel GP's over the past years. Neither did we know to what extent the EHR data of the sentinel GPs are fit for research purposes. This exploitability should be evaluated by data quality criteria and in reference to features of Belgian general practice and the (health) policy system.

Belgium is known for its high rate of home visits and out-of-hours care in general practice [11]. The policy system has evolved towards a federal model composed of communities with specific cultural identities and different languages. The Dutch speaking community (northern part of Belgium) and the French speaking community (southern part of Belgium) have jurisdiction over matters that are linked to people rather than territory, such as health and social support insofar as they are not part of the social security system. The sentinel network is supported by the two concerned public health Ministries, having set their own health priorities based on the health needs of their communities. This explains the importance of a random sample of sentinel GPs in both communities.

In order to deliver data fit for research, the sentinel GPs should comply with some quality criteria on the management of patient information. For instance, free text notes might be vital in daily patient care, for research purposes they are rather useless. Easy retrievability of drug treatment codes and diagnostic codes were expressed in rate of use of the prescription module and rate of use of the system-provided (uniform) subject headings for symptoms and diagnoses. A government certification requirement for EHR systems is that subject headings should be exchangeable to codes of the International Classification of Primary Care (ICPC). A third criterion measures the rate by which data of patients seen in home visits are recorded in the EHR system. Omission of cases from home visits could bias study results as they often concern aged people. Due non-existing valid measures of the fit of EHR data for research, we copied these criteria from a survey of a random sample of Belgian GPs by the Belgian ResoPrim project (2003-2008) involving co-author VVC [12,13].

Our research questions were: How many sentinel GPs were using a certified EHR system the last year they participated and what specific software package? To what extent is this use associated with the key characteristics of the sentinel GPs? What is the level of compliance with three data quality criteria? To what extent is this compliance associated with the key characteristics of the sentinel GPs and the EHR software package?

## Methods

This study was part of a larger research project, i.e. the postal survey held yearly among all participating sentinel GPs. The data of this study were collected by five annual surveys between 2005 and 2009. All data concern the last year of participation, apart from two variables concerning 2009. For the variables practice organisation (solo/group), use of a (certified) EHR system (yes/no), EHR software package and frequency of recording of home visits in EHR, data from the last year of participation were used. Data on frequency of use of prescription module and subject headings are from spring 2009. No validation of the main outcome measures, three data quality criteria, was available. Frequency of compliance to data quality criteria were measured by Likert scales [never (1), sometimes (2), usually (3), always (4)]. A shortened version of the questionnaire can be viewed in additional file 1. For each quality criterion a dummy was constructed with a value of 1 if the rate of use equalled or exceeded the median value.

Bivariate correlation between the three quality criteria compliance rates was expressed in Spearman's r. Odds ratio's were calculated to explore the relation between independent variables, i.e. four key characteristics of the sentinel GPs (age group, gender, community, practice organisation), EHR software package and compliance with data quality criteria. Four key characteristics were included in a multivariable logistic regression with the use of a certified EHR system as a dependent variable. The compliance with three quality criteria was equally measured by multivariable logistic regression models. Only the top three of EHR software packages was included in the models by constructing a categorical variable with the remaining certified EHR software packages as reference group.

## Results

Data were collected from 221 sentinel GPs. Median age was 52 years (interquartile range, 47-58), 67% of the GPs were men, 53% were living in the Dutch community and 28% were organised in a group practice. More than half (55%) of them participated in the surveillance without interruption from 2005 onwards, i.e. all five years and 78% of the GPs were participating in 2009. Three of 215 GPs having participated between 2005 and 2008 did not respond to the survey (participation rate in 2009 was still unknown in November 2009).

In their most recent participation year, 91% of the Dutch and 63% of the French speaking sentinel GPs (78% overall) were using a certified EHR system. The odds for using a certified EHR system were much higher in group practices, in the Dutch speaking community and among younger GPs (Table 1).

**Table 1:** Gender, age, language community and practice organisation as determinants of the use of certified EHR systems: results from a multivariable logistic regression (N = 221)

	OR	95% CI	P
**Gender**			
Women	1		0.120
Men	2.01	0.83-4.86	
**Age**			
< median	2.43	1.05-5.61	0.037
≥ median	1		
**Community**			
French	1		0.000
Dutch	5.43	2.45-12.02	
**Practice organisation**			
Solo	1		0.003
Group	9.52	2.15-42.17	

Abbreviations: OR: Odds Ratio; CI: confidence intervals; P: statistical significance.

Significant P-values are in bold.

Sixteen (out of 20) certified EHR systems were used. The EHR software package was strongly related to the community. To include (almost) 80% of the sentinel GPs in both communities, six EHR systems were needed on the French side and four on the Dutch side (Table 2). Only one EHR system, Health One^©^, was used by a comparable number of sentinel GPs in both communities.

**Table 2:** Most frequently used certified EHR systems by language community of the sentinel GPs (N = 173)

	French speaking sentinel GPs		Dutch speaking sentinel GPs	
	**N = 51 (of 65)°**	**Cumulative %**	**N = 85 (of 108)°**	**Cumulative %**

Windoc^©^	(1)*		35	32.4
Medidoc^©^	(4)		22	52.8
Health One^©^	18	27.7	18	69.4
Accrimed-2000^©^	(0)		10	78.7
Epicure^©^	9	41.5	(1)	
Mediwin^©^	7	52.3	(0)	
Medigest^©^	6	61.5	(3)	
Socrate Medical^©^	6	70.8	(0)	
Le Généraliste^©^	5	78.5	(0)	

° Numbers refer to the total group size and, between brackets, the number of GPs included in the cumulative proportion of (nearly) 80% of the users.

* Number of users of less frequently used EHR systems, i.e. not included in the cumulative proportion of (nearly) 80% of the users, are between brackets.

Of the three criteria, the lowest use was made of the list of uniform subject headings provided by the EHR system (Table 3). Half of the sentinel GPs was always using the prescription module of the EHR system. The proportion of sentinel GPs (14%) never recording home visits in the EHR was small.

**Table 3:** Compliance rates with three data quality criteria

	Use of subject headings		Use of prescription module		Recording of home visits in EHR	
	**N = 141**,	**median = 2**	**N = 141,**	**median = 4**	**N = 186**,	**median = 3**
	**N**	**%**	**N**	**%**	**N**	**%**

Never (1)	47	33.3	27	19.1	26	14.0
Sometimes (2)	38	27.0	10	7.1	61	32.8
Usually (3)	32	22.7	33	23.4	43	23.1
Always (4)	24	17.0	71	50.4	56	30.1

The highest correlation (all p < 0.001) was found between the use of uniform subject headings and prescription module (Spearman's r = 0.516) and the lowest between recording of home visits and, respectively, prescription module (Spearman's r = 0.283) and uniform subject headings (Spearman's r = 0.346).

The compliance with three criteria was univariately significantly related to practice organisation and community but not to age and gender. The odds of using uniform headings were higher in group practices than in solo practices [odds ratio (OR) 2.22; 95% confidence interval (CI) 1.01-4.90]. The prescription module was more frequently used by Dutch speaking sentinel GPs than by the French (OR 1.99; 95% CI 1.00-3.95). Multivariate logistic regression showed that the odds for using subject headings were higher among users of Health One^© ^and Medidoc^© ^(Table 4).

**Table 4:** EHR software package as determinant of compliance with three data quality criteria: results from multivariable logistic regression analyses

EHR software package	Use of subject headings (N = 134)*			Use of prescription module (N = 134)*			Recording home visits (N = 173)*		
	
	OR	95%CI	P	OR	95%CI	P	OR	95%CI	P
Other system	1			1			1		
Health One^©^	4.02	1.21-13.34	**0.023**	2.34	0.88-6.25	0.089	1.01	0.44-2.31	0.982
Windoc^©^	0.82	0.28-2.36	0.713	0.62	0.22-1.76.	0.373	0.58	0.24-1.42	0.235
Medidoc^©^	4.15	1.03-16.69	**0.045**	1.51	0.49-4.68	0.475	0.86	0.33-2.25	0.765

Abbreviations: OR: Odds Ratio; CI: confidence intervals; P: statistical significance

Significant P-values are in bold.

* In the three multivariable models, we adjusted for gender, age (under median and median or higher), community and practice organisation. GPs with a compliance rate equalling or exceeding the median value (1) were compared to GPs with a compliance rate under the median value (0).

## Discussion

This study shows that a vast majority of the sentinel GPs was using a certified EHR system in the last year of participation between 2005 and 2009. This is largely due to the high use in the Dutch speaking community compared to the French. Of all 16 certified EHR systems in use, only one (Health One^©^) ranked in the top three in both communities. Overall, the prescription module was always used and home visits were usually recorded. Uniform subject headings were only sometimes used and the compliance with this quality criterion was exclusively related to the EHR software package in use.

Our study throws a new light on the quality of EHR data for research purposes by showing the relation with the EHR system in use. A previous study in Belgian general practice also found that data quality indicators, i.e. the completeness of drug treatment data, were quite different according to the (anonymous) EHR system [8]. As far as we know, the relation between quality of EHR data and software package had only once been examined [14]. This study compared the medication registration after an upgrade of the EHR software package and found an improved completeness.

Our main outcome measures were not validated in previous studies. So far, characteristics of EHR data affecting their usefulness for research were described only in a tentative way but our criteria are very similar [15].

Our assessment of compliance with data quality criteria was based on self reports and possibly overestimated by memory effects and social desirability. We did not observe actual practice patterns of GPs nor did we study the content of EHR, i.e. the correctness and completeness of EHR morbidity data. Yet, the quality of extracted EHR data in Belgian general practice was assessed in the ResoPrim project (2003-2005). Its preliminary conclusion was that it is still a long way from using routinely collected EHR data for epidemiological studies, mainly due their incompleteness and less to their incorrectness [9].

Our findings are comparable with results of three Belgian studies. The proportion of sentinel GPs using a certified EHR system (78%) is high compared to the proportion of certified and full time working Belgian GPs that were entitled in 2005 to receive a lump sum for using a certified EHR system (66%) [16]. A survey in a random sample of 448 Belgian GPs in 2006 showed that 70% was using an EHR system, 76% of the Dutch speaking GPs and 62% of the French speaking GPs [17]. This survey showed identical EHR systems in the top three among Dutch and the top five among French speaking GPs with equally only Health One^© ^in common. Finally, the survey from which we copied the three quality criteria showed similar results on the frequency of use of uniform subject headings and the prescription module among 1028 Belgian GPs [13]. Only the proportion of GPs recording home visits usually or always in their certified EHR system was higher among sentinel GPs compared to the random sample (51% versus 41%, p < 0.05).

As expected, no significant relation was found between EHR system software and the level of recording of home visits, a practice presumed to be completely GP related. This criterion has a weak correlation with the two other criteria on diagnosis and therapy, key components of patient data. The high use of the prescription module is not surprising: a systematic review of studies on EHR data quality in primary care confirmed that prescription data have the highest rate of recording [18]. The attribution of uniform subject headings to symptoms and diagnoses is only fully achieved by a minority of sentinel GPs. Two EHR systems appear to improve the use of the provided uniform subject headings. It seems self-evident that the human-computer interfaces of Medidoc^© ^and Health One^© ^are worth being studied and, possibly copied by other EHR software package vendors. Also, Health One^© ^seems the first choice EHR software package in the transition process to (semi-) automatic data extraction as it is used both by French and Dutch speaking sentinel GPs. Yet we can not exclude that EHR systems have their own user profiles, independent of the system or not. As a matter of fact, Medidoc^© ^is used by Intego, an EHR-based registration network of Dutch speaking GPs, but only four sentinel GPs appeared to be also trained participants in Intego [19].

From lessons learnt about the use of EHR for research (in networks), we retain several, multifaceted approaches to increase the quality of data [6]. These include starting with relative simple research questions, use of pop-ups to force correct data entry, motivation of network members and support from peers and academics, financial incentives and so on. A literature review on methods of data quality improvement in general practice concluded that empirical knowledge is still missing [20]. Although there seems to be ample space for improvement, criteria and acceptable levels of data quality are lacking. Quality assurance interventions were not described in detail but ranged from individual feedback with or without a comparison with group performance, self-auditing, group meetings to training sessions.

This study confirms that the network of sentinel GPs is highly representative of Belgian general practice, including the use of a high variety of certified EHR systems. This representativeness has a high price since developing and implementing data extracting modules for all certified EHR systems in use seems unfeasible due high costs. The challenge lies in the reconciliation between a representative network, covering both communities and high EHR data quality.

## Conclusions

This survey shows that current use of certified EHR systems among Dutch speaking sentinel GPs was much higher compared to French speaking GPs. The level of compliance with three data quality criteria was mixed and partly related to the EHR software package. The challenge is to progress towards a network of sentinel GPs delivering care-based data that are (partly) extracted from well performing EHR systems and still representative for Belgian general practice.

## Competing interests

The authors declare that they have no competing interests.

## Authors' contributions

N Boffin participated in the design of the study, performed the statistical analysis and drafted the manuscript. NB, KV and VVC made substantial contributions to the study design, data interpretation and statistical analysis, and helped to draft and revise the manuscript. All authors read and approved the final version.

## Pre-publication history

The pre-publication history for this paper can be accessed here:

http://www.biomedcentral.com/1471-2296/11/50/prepub

## Supplementary Material

Additional file 1 **Questionnaire**. The file contains a shortened version of the questionnaire sent to the GPs. Click here for file
