# Supplementary material for: Readiness of the Belgian network of sentinel general practitioners to deliver electronic health record data for surveillance purposes: results of survey study
Source: BMC Fam Pract. 2010 Jun 25;11:50. doi: 10.1186/1471-2296-11-50 (PMC2910665; doi:10.1186/1471-2296-11-50)
Supplement: Additional file 1 — Questionnaire. The file contains a shortened version of the questionnaire sent to the GPs. [file 1471-2296-11-50-S1.DOC]

# Questionnaire*

- Do you keep electronic health records (EHR) of your patients? (Yes/No)
- In your daily practice do you use the subheadings provided by the EHR software to record symptoms, complaints and diagnoses?
  - Never
  - Sometimes
  - Usually
  - Always
- In your daily practice do you use a prescription module in order to find prescribed medication in the EHR?
  - Never
  - Sometimes
  - Usually
  - Always
- Do you record home visits in the EHR?
  - Never
  - Sometimes
  - Usually
  - Always

* Five additional questions concern gender, age, practice organisation, credentialing, Internet access and palliative care practices.
